# Performance of High-Workability Mortars Incorporating Metakaolin as a Partial Cement Replacement

**DOI:** 10.3390/ma19081558

**Published:** 2026-04-14

**Authors:** Natividad Garcia-Troncoso, Mohamad Alnasser, Chenmeng Zhang, Dan V. Bompa

**Affiliations:** 1 Faculty of Engineering in Earth Sciences, Escuela Superior Politécnica del Litoral (ESPOL), Campus Gustavo Galindo, Km. 30.5 Vía Perimetral, Guayaquil 090902, Ecuador; 2 Centro de Investigación y Desarrollo en Nanotecnología, Escuela Superior Politécnica del Litoral (ESPOL), Campus Gustavo Galindo, Km. 30.5 Vía Perimetral, Guayaquil 090902, Ecuador; 3 School of Engineering, University of Surrey, Guildford GU2 7XH, UK; nasser@kabrillc.com (M.A.); chenmeng.zhang@surrey.ac.uk (C.Z.)

**Keywords:** metakaolin, high-workability mortar, supplementary cementitious materials, durability-related transport properties, carbon footprint

## Abstract

This study investigates the effect of metakaolin (MK) as a partial replacement of cement (CEM I) in high-workability mortars, with emphasis on fresh-state behaviour, mechanical properties, microstructural development, and carbon footprint implications. Mortars were produced with MK replacement levels ranging from 0 to 50% by mass of binder, under a constant water-to-binder ratio and fixed superplasticiser amount. Fresh-state results showed that increasing MK content reduced flowability due to its high fineness; however, high workability was maintained for replacement levels up to 20%. At 28 days, MK replacement up to 10% retains approximately 90–95% of the control compressive and flexural strength, whereas higher replacement levels lead to gradual strength reductions (to ~55–60% at 50% MK), despite comparable early-age strength gains across all mixes. Durability-related indicators demonstrated reduced water absorption and capillary uptake at moderate MK contents (approximately 20–30%), indicating refined pore structure and reduced pore connectivity. Microstructural analyses using SEM, TGA, and XRD confirmed effective portlandite consumption and the formation of dense C–A–S–H-type hydration products at moderate MK replacement levels, whereas excessive MK contents resulted in unreacted MK. A comparative carbon footprint assessment showed that MK incorporation leads to proportional reductions in embodied CO_2_ emissions, with replacement levels of 10–20% providing the most favourable balance between mechanical performance, durability, and environmental benefit. Therefore, the results demonstrate that MK can be used as a supplementary cementitious material for producing low-carbon, high-workability mortars.

## 1. Introduction

The cement and concrete industry are under increasing pressure to decarbonise in response to global climate targets. Cement production accounts for approximately 7–8% of global anthropogenic CO_2_ emissions, primarily due to the calcination of limestone and the high thermal energy demand of clinker manufacture [1,2]. Achieving meaningful emissions reductions requires targeting substantial reductions in clinker content [3]. Partial replacement of cement with supplementary cementitious materials (SCMs) is an effective and readily implementable approach [4,5]. Metakaolin (MK) is a promising SCM for low-carbon cementitious systems. MK is produced by the controlled calcination of kaolinitic clays at 650–800 °C, resulting in an amorphous aluminosilicate material rich in reactive silica and alumina [6]. When incorporated into cement-based systems, MK modifies the hydration of cement primarily through a coupled filler and pozzolanic effect [7].

Compared with plain cement systems, at early concrete age, MK accelerates hydration by promoting the reactions of silicate and aluminate phases, shifting the silicate peak to earlier times, increasing sulphate depletion, and enhancing ettringite formation [8,9]. MK reacts with portlandite to form secondary calcium silicate hydrate (C-S-H) and calcium aluminosilicate hydrate (C-A-S-H), lowers the intensity or amount of residual CH, increases chemically combined water, and shifts the hydrate assemblage towards alumina phases [10,11,12]. This is accompanied by compositional modification of the main binding gel, including lower Ca/Si and higher Al incorporation in C-A-S-H [10,12]. MK also promotes the formation of additional hydrate phases, which contribute to matrix densification and capillary void filling [13,14,15]. These hydration changes are consistently associated with pore refinement and increased tortuosity, although total porosity does not always decrease monotonically with increasing replacement level [16,17]. In blended systems MK reduces total porosity and produces a more homogeneous and contiguous solid matrix with lower water mobility [18].

MK replacement levels of cement by around 5–20% generally increase compressive, tensile and flexural strengths relative to control mixes [19,20,21,22]. A 20% replacement increased 28-day compressive strength from 62.3 MPa to 74.9 MPa in one study on self-compacting concrete [23], whilst a 10 wt.% replacement led compressive strengths of 48.4, 71.2 and 76.1 MPa at 7, 28 and 56 days in another self-compacting concrete [10]. Compressive strength gains of about 10–15% over the control have also been reported [13], and even low-grade MK at high replacement can maintain comparable 28-day strength, with up to 40 wt.% replacement reported in one study [12]. In aged Portland cement, MK also improved physical and mechanical performance by restoring hydration reactivity, with 5–10% replacement significantly improving 7- and 28-day strength [9]. High MK replacement levels (>30–40%) can lead to reductions in early-age strength and increases in porosity due to dilution effects and incomplete hydration under standard curing [24,25].

MK generally reduces the flowability of cementitious mixes and increases fresh-state water and admixture demand because of its high specific surface area, angular or flaky particle morphology, and consequent increase in internal friction and water adsorption [9,10,13,26]. For large replacement levels, these effects become more critical, particularly when rheological stability is essential to ensure uniform placement without segregation [27,28,29]. In terms of concrete fresh properties, these effects are usually expressed as lower slump flow, longer flow times and higher superplasticiser demand. For example, in self-compacting concrete, increasing MK slightly reduced slump flow from 690 to 675 mm, increased T50 from 2.3 to 3.3 s, and increased V-funnel time from 4.8 to 5.9 s [10]. Similarly, MK increased the water demand or increased the superplasticiser content required to achieve a given slump in conventional concrete [13,22]. MK also tends to increase cohesion and viscosity, which can be beneficial for stability [30]. In fresh mortar systems, below 15% replacement, MK had little effect on yield stress, whereas above this level, it substantially increased the yield stress; plastic viscosity increased steadily with MK content, indicating a clear tackifying effect [31].

Within the broader group of low-clinker cements, MK represents the high-reactivity component in the calcined clays cements and supports the potential for large-scale decarbonisation [7,32]. In these systems, MK contributes to early porosity refinement and strength gain through carboaluminate formation, while later-age strength becomes less sensitive to MK content [33]. In MK–limestone systems, MK stimulates early alite hydration, sustains heat release, promotes ettringite formation, and strongly enhances hemicarboaluminate and monocarboaluminate formation through reaction with the limestone [34].

Developments in low-carbon concrete research emphasise the need for integrated performance-based evaluation frameworks combining structural performance, durability, and environmental impact [35,36]. Life-cycle assessment studies demonstrate that clinker substitution can lead to substantial embodied carbon reductions but also show that these benefits are highly sensitive to binder composition, material variability, and assumptions regarding service life [37]. Probabilistic approaches have highlighted the importance of accounting for uncertainty in embodied carbon assessments in comparative studies [38].

This paper aims to define a practical threshold map for optimum MK replacement, rather than a single optimum, particularly since the optimum amount depends on the requirements of the intended application, in line with performance-based approaches. To achieve this, a wide MK replacement range of 0–50% by binder mass was investigated, capturing the transition from beneficial incorporation in cement blends to calcium-limited or dilution-dominated responses of MK. Unlike most existing studies, which achieve target fresh properties by increasing water or superplasticiser amount, this work adopts a controlled-variable design with constant parameters across all mixes, thereby isolating the intrinsic effect of MK substitution. The study integrates fresh-state response, compressive strength at 7, 28 and 90 days, 28-day flexural strength, water absorption and capillary sorptivity, SEM–TGA–XRD microstructural assessments, and environmental impact within a single experimental programme, whereas previous studies have generally addressed only subsets of these properties.

## 2. Materials and Methods

The present study adopts a controlled-variable experimental design, in which only the cement replacement level by MK is varied, while all other parameters, aggregate content, water content, admixture amount, curing conditions, and testing procedures, are held constant [17,29]. The work therefore presents an integrated assessment of performance and sustainability and establishes practical limits for MK incorporation in low-carbon, high-workability mortars for overlay or self-levelling applications that require placement in vibration-free environments.

### 2.1. Constituent Materials

A conventional cement conforming to BS EN 197-1 [39], strength class of CEM I 52.5R, was used as the primary binder. According to the manufacturer’s technical datasheet, the cement has a clinker content of at least 95%, a typical initial setting time of 135 min, a typical final setting time of 220 min, and minimum compressive strengths exceeding 30 MPa at 2 days and 52.5 MPa at 28 days [40]. Based on literature data, Whitestar CEM I 52.5R has a specific gravity of 3.15 g/cm^3^ and a specific surface area of 0.42 cm^2^/g [41,42,43]. Published data for comparable CEM I 52.5R cements indicate a median particle size, D50, of about 7.0 μm [44] and an average particle diameter of about 17 μm [45]. A representative oxide composition of the cement includes 63.5% CaO, 19.9% SiO_2_, 5.2% Al_2_O_3_, 3.4% Fe_2_O_3_, 1.7% MgO, 0.8% Na_2_O, and 0.7% K_2_O and a loss of ignition of around 1.07 [46].

**Figure 1:**
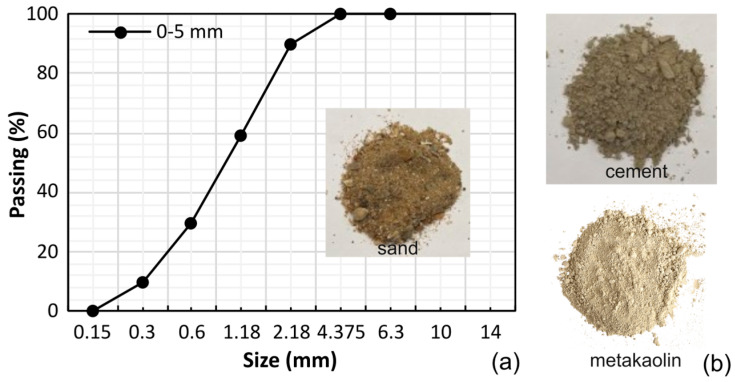
(**a**) Sieve analysis results and view of the fine aggregate. (**b**) Binders.

MK was used as an SCM to partially replace cement by mass of binder. The selected product was Argical M-1000, a commercially available high-purity MK derived from calcined kaolinitic clay, with manufacturer specifications provided in the product datasheet [47]. This material is characterised by a high amorphous aluminosilicate content, fine particle size, and plate-like morphology, which together contribute to high pozzolanic reactivity but also increased water demand. The product has a reported chemical composition of approximately 55% SiO_2_, 40% Al_2_O_3_, 0.8% K_2_O + Na_2_O, 1.4% Fe_2_O_3_, 1.5% TiO_2_ and 0.3% CaO + MgO, with a loss on ignition of 1% [47]. With respect to particle size, the sieve analysis provided by the manufacturer indicates that 95% of the material is finer than 80 μm, with a specific gravity of 2.4 g/cm^2^ and a specific area of 17 m^2^/g. A natural siliceous fine aggregate with a nominal maximum particle size of 5 mm was used in all mixes. The aggregate complied with BS EN 12620 requirements for aggregates for concrete [48]. Aggregate properties were kept constant across all mixtures to isolate the effect of binder composition on fresh and hardened properties. The particle size distribution of the fine aggregate, determined in accordance with BS EN 933-1, is reported in Figure 1 [49].

Potable tap water conforming to BS EN 1008 was used for all mixes [50]. Water temperature at the time of mixing was maintained at laboratory ambient conditions (20 ± 2 °C). A polycarboxylate ether (PCE)-based high-range water-reducing admixture Flowaid SCC) suitable for high-workability mortars was used to achieve the target flowability at constant water content [51]. The admixture conformed to BS EN 934-2 [52].

### 2.2. Mix Design and Mixing Procedure

The experimental matrix comprised six formulations based on binary CEM-MK binder systems, in which MK was used as a partial replacement of CEM I cement by mass of total binder. Replacement levels of 0, 10, 20, 30, 40, and 50% were selected, and the corresponding mixtures were denoted as CEMxx_MKyy, where xx is the cement percentage and yy is the MK percentage of the total binder mass. For example, CEM50_MK50 indicates a mix with equal quantities of CEM and MK by mass (Table 1). The MK replacement range was chosen to cover from conventional low-level MK incorporation to high replacement levels approaching the practical upper bound for cement-based systems [17,23]. All other mix parameters were held constant. The sand-to-binder ratio was fixed at 2.5, consistent with proportions typically adopted for highly flowable mortars [29].

The CEM100_MK0 control mix targeted a 28-day compressive strength of 60 MPa, proportioned using a powder-type self-compacting design approach with w/b = 0.60 and fines/binder = 2.5 [53,54]. This approach is often adopted in SCM studies, as it prevents confounding effects arising from simultaneous changes in water content, paste volume, or admixture content and allows performance trends to be attributed directly to binder substitution [4]. Maintaining a constant water-to-binder ratio is essential when assessing strength development and transport-related durability indicators [55].

All mortar mixtures were prepared using a 5 L laboratory-scale rotary pan mixer equipped with paddle action. The mixing protocol was strictly standardised across all batches, as previous studies have demonstrated that variations in mixing order, duration, and shear intensity affect material properties [7]. Prior to water addition, the fine aggregate, cement, and MK were introduced into the mixer and dry-mixed for a duration of 2 min. This initial dry blending stage was implemented to promote uniform distribution of the binder components and to reduce the formation of MK agglomerates. Following dry mixing, approximately 80% of the total mixing water, pre-dissolved with the full amount of PCE-based superplasticiser, was added gradually over approximately 30 s while the mixer operated at low speed. Pre-dissolving the admixture in the mixing water ensured uniform distribution of the dispersing agent. The remaining 20% of the water was subsequently introduced over approximately 60 s as the mixing speed was increased to a medium setting, allowing progressive wetting of the dry constituents and controlled development of paste. After completion of water addition, mixing was continued at medium speed for a further 2.5 min. During this stage, the internal walls of the mixer were manually scraped at regular intervals of 30 s to reincorporate adhered paste and ensure complete material homogenisation [29]. A final high shear mixing stage of 1 min was then applied to promote full dispersion of remaining particles. All mixtures were produced at laboratory ambient temperature (20 ± 2 °C), and mixing commenced immediately prior to fresh-state testing and specimen casting to minimise the influence of early hydration and thixotropic rebuilding on workability measurements.

After completion of the mixing, the fresh mortar was subjected to consistency assessment using the flow table method stipulated in BS EN 1015-3 and then cast into moulds for hardened-state testing [56]. For compressive strength testing, twelve 50 × 50 × 50 mm cube specimens were cast per mix. For flexural strength testing, three 40 × 40 × 160 mm prism specimens were cast per mix. Cube and prismatic geometry and testing procedures are consistent with the principles of BS EN 1015-11 [57]. Mortar placement was performed without the application of external vibration. This approach was adopted to ensure that the measured fresh state behaviour, and the resulting hardened performance were representative of highly flowable mortars. Each mould was filled in a continuous operation to avoid cold joints with the mortar poured from a height of 50 mm from the mould’s upper side. The mould surface was then levelled using a steel trowel, and all specimens were immediately covered with impermeable plastic sheeting to prevent evaporation during the first 24 h. All specimens were stored during the first 24 h under controlled laboratory conditions (20 ± 2 °C). Then, specimens were demoulded and transferred to water curing at 20 ± 2 °C until testing at 7, 28 or 90 days. At each age, at least three specimens were tested per mix, with remaining samples used for additional verification tests if required.

### 2.3. Fresh-State, Hardened-State and Water Absorption Testing

**Figure 2:**
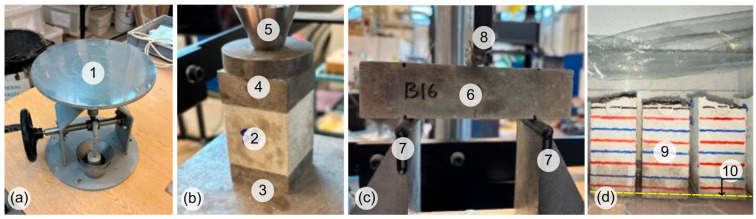
(**a**) Flow table apparatus for fresh mixes. (**b**) Compression testing setup for cubes. (**c**) Three-point bending setup for prisms. (**d**) Capillary absorption procedure (notes: (1) flow table, (2) cubic specimen, (3) support steel plate, (4) top steel plate, (5) load application and hinge, (6) prismatic sample, (7) supports, (8) load application strip, (9) half prismatic specimens with gradation, (10) water level for capillary absorption showed in dashed yellow line).

Fresh properties were quantified through the flow table method, as it is a widely adopted standardised procedure for mortars [56] (Figure 2a). The truncated conical mould was positioned centrally on the flow table and filled in a single lift; the mortar was lightly tamped with a trowel to remove entrapped air, and excess mortar was struck off flush with the mould rim. After approximately 15 s, the mould was lifted vertically to allow free spreading. The table was then subjected to 25 jolts over 15 s, corresponding to the prescribed standard jolt regimen. The spread diameter was measured along two orthogonal directions, and the mean of the two readings was reported as the flow diameter. In the experimental program, the flow test was completed within 5 min after mixing to reduce the influence of early stiffening on the measured spread.

Compressive strength was determined at 7, 28 and 90 days using 50 mm mortar cubes produced for each CEM-MK mix, with at least three replicate specimens tested per age and mix, and four specimens tested where available (Figure 2b). Testing was conducted in accordance with BS EN 1015-11, which defines the requirements for loading arrangement, specimen alignment and reporting for hardened concrete specimens and is widely adopted for cementitious composites where cube geometries are used [57]. The compression apparatus comprised a calibrated testing machine with a 1 MN load cell, operated under displacement control, and loading was applied at a constant rate equivalent to 0.5 mm/min until failure under monotonic compression. The maximum load at failure, denoted as F_max_, was recorded automatically, and the cube compressive strength (f_c_) was calculated using the expression f_c_ = F_max_/A, where A is the loaded cube area. For each mix and age, the mean compressive strength and standard deviation were reported.

Flexural strength was measured on 40 × 40 × 160 mm prisms under three-point bending [57]. The support span was 100 mm, with supporting and load application rollers diameters of 10 mm and 2 mm, respectively, and the load was applied under deflection control at 0.2 mm/min until failure (Figure 2c). Flexural strength was calculated using the codified expression for three-point bending of prismatic specimens, f_f_ = (1.5 × F_max_ × L)/(b × d^2^), where (L) is the span length and (b) and (d) are the specimen width and depth. Immediately after flexural testing, the broken prism halves were retained for subsequent water absorption testing to ensure that durability-related metrics were obtained from specimens cured and stored identically to those used for mechanical performance.

Water absorption by capillary uptake was carried out the next day after the 28-day strength assessments using the guidance given in BS EN 13057 [58]. Capillary water absorption was measured to characterise early-age absorption-driven ingress, which is frequently described by sorptivity (S), derived from the linear relationship between cumulative absorption and the square root of time for capillary-dominated uptake [59,60]. The tests were conducted on broken prism halves with approximate dimensions of 40 × 40 × 80 mm. Specimens were oven-dried at 105 °C for 24 h and then cooled prior to testing, maintaining a consistent initial moisture state. Measurements were taken at 12 min, 30 min, 1 h, 2 h, 4 h and 24 h to capture the rapid early uptake and the subsequent evolution. The samples were submerged 3 mm in the water; thus, water uptake was through the sample bottom and around 3 mm of their height (Figure 2d). Cumulative absorption was assessed from the normalised mass increase, and sorptivity was obtained as the slope of the capillary uptake versus square root of time, for two stages: stage one included the initial time considered up to 1 h, and the second stage for a time period of 1–24 h [61]. At least two specimens per mix were tested to verify repeatability of sorptivity trends.

### 2.4. Microstructural Analysis Methods

The present methodology integrates: (i) X-ray Diffraction (XRD) for crystalline phase identification and quantification via whole-pattern fitting/Rietveld principles [62], (ii) Thermogravimetric Analysis (TGA) and Derivative TG (DTG) to independently quantify chemically bound water and key decomposition events (e.g., portlandite and carbonates) [63], and (iii) Scanning Electron Microscope with Energy-Dispersive Spectroscopy (SEM/EDS) to resolve microstructural heterogeneity and verify the morphology/chemistry of phases at the microscale [64]. All analyses were performed on hardened binder/mortar specimens after mechanical testing or at predefined ages [65]. For the microstructural characterisation, the specimens were approximately 164 days old. For XRD and TGA/DTG analyses, samples were ground to a maximum particle size of 45 µm, whereas SEM/EDS observations were conducted on fragments with a maximum size of 5 mm. To prevent further hydration and preserve the microstructural state, the samples were immersed in ethanol and dried in desiccators for 48 h [66]. After completing the procedure, the samples were stored in a moisture-free chamber to avoid alterations. The duration of the three tests (XRD, TGA/DTG, and SEM/EDS) was approximately 2 h per sample. During this period, the remaining specimens were kept inside the controlled chamber to prevent any changes induced by external environmental conditions.

**Figure 3:**
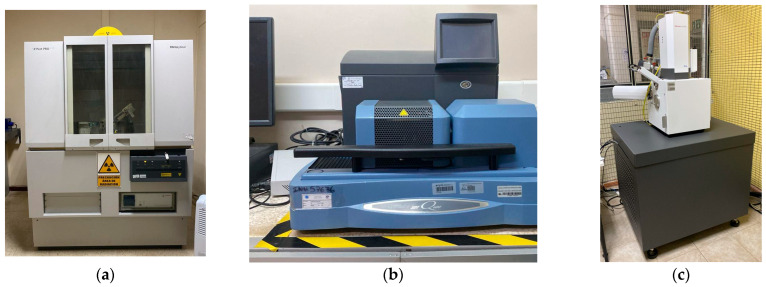
(**a**) XRD equipment. (**b**) TGA equipment. (**c**) SEM equipment.

X-ray Diffraction (XRD) was performed using a PANalytical X’Pert PRO diffractometer (Malvern Panalytical Ltd., Malvern, Worcestershire, UK) (Cu/Co tube configuration; instrument label PW 3040/60), operated with Co radiation and an X-ray tube setting of 40 kV and 20 mA (Figure 3a). Patterns were acquired from 5.0251° to 79.9751° (2θ) with a 0.050° step size and 20 s counting time per step. Diffractograms were processed and quantified using X’Pert HighScore Plus (PANalytical; software v2.2d/2.2.4, as recorded during analysis), which supports phase identification and whole-pattern fitting approaches consistent with Rietveld-type refinement logic [62]. Quantitative outputs were reported for crystalline phases (e.g., residual clinker minerals, portlandite, carbonates, and other crystalline hydrates where detectable) [64].

Thermogravimetric Analysis (TGA) was conducted using a TA Instruments SDT Q600 simultaneous thermal analyser (TGA/DSC capability) (Figure 3b), enabling measurement of mass loss and its derivative (DTG) over controlled heating. The thermal program used a Nitrogen (N_2_) atmosphere with a heating ramp of 10 °C/min, from 20 °C to 900 °C, recorded in the instrument method file used during testing, ensuring coverage of dehydration/dehydroxylation and decarbonation events relevant to hydrated cementitious binders [63]. Each sample introduced into the SDT apparatus had an approximate mass of 10.5 mg. Mass-loss regions were interpreted using established cementitious binder conventions: dehydration of C-A-S-H/AFt/AFm at low to mid temperatures, portlandite dehydroxylation, and carbonate decarbonation at higher temperatures [66]. For SCM-containing systems, portlandite consumption trends are directly relevant to estimating the extent of pozzolanic reaction and validating XRD-observed CH reduction. The TGA results were used to validate the phase evolution inferred from XRD.

Scanning Electron Microscopy (SEM) was performed using a Thermo Fisher Scientific Axia ChemiSEM (Hillsboro, OR, USA) (Axia HV/LoVac platform) (Figure 3c), enabling high-resolution imaging and Energy-Dispersive Spectroscopy (EDS) for elemental microanalysis. SEM-EDS is essential in cementitious materials because it resolves the spatial distribution of hydrates, residual anhydrous particles, and reaction rims that cannot be inferred reliably from bulk methods alone [67]. For imaging and EDS, the instrument was operated at an accelerating voltage of 20 kV (spot size 3.0), with acquisition under high-vacuum conditions, as specified for the measurement campaign. Polished cross-sections were used to enable backscattered electron contrast and quantitative compositional assessment of phases; imaging was complemented with EDS point analyses and/or maps to confirm phase chemistry. SEM observations were explicitly used to (i) verify whether phases identified by XRD were present in the expected morphology and location and (ii) interpret reaction progress and heterogeneity that can bias bulk quantification if not recognised.

### 2.5. Carbon Footprint Assessment

The present study addresses this gap by embedding the embodied CO_2_ assessment directly within the experimental program, using the same mix designs and performance data discussed in preceding sections. This integrated approach enables an evaluation of how clinker replacement translates into emissions savings. Embodied CO_2_ emissions were quantified using a cradle-to-gate approach (stages A1–A3), consistent with current best practice for comparative binder assessments [68]. The system boundary includes raw material extraction, processing, and manufacturing of all binder constituents, while excluding transport, placement, and use-phase emissions, which are assumed identical for all mixes and therefore do not affect relative comparisons. For each mix, the total embodied CO_2_ per cubic metre of mortar (Table 2), ${CO}_{2,|mix}$, was calculated with Equation (1), where $m_{i}$ is the mass of constituent *i* (kg m^−3^) and ${EF}_{i}$ is its corresponding embodied-carbon emission factor (kg CO_2_ /kg).

(1) $${CO}_{2,|mix}=\sum_{i}m_{i}\cdot {EF}_{i}$$

Emission factors were taken from peer-reviewed literature and widely used databases [36]. CEM was assigned an emission factor of approximately 0.90 kg CO_2_ kg^−1^, MK values ranged between 0.25 and 0.35 kg CO_2_ kg^−1^, depending on calcination assumptions, following allocation approaches commonly adopted in cement and concrete LCAs [3]. All non-binder constituents (fine aggregate, water, and superplasticiser) were included using literature-based emission factors; however, their contribution to total embodied CO_2_ remained constant across mixtures.

## 3. Experimental Results and Discussion

### 3.1. Fresh Properties

**Figure 4:**
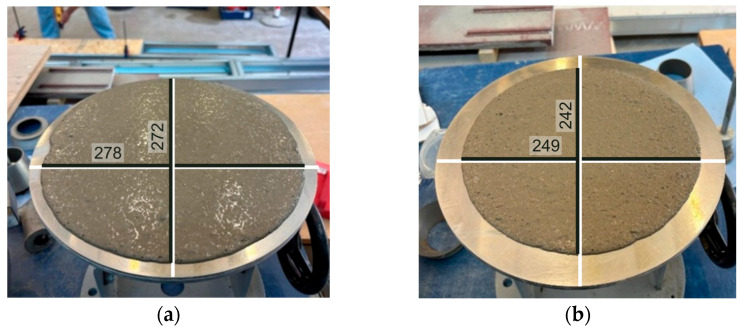
Flow diameter for selected CEM-MK mixes: (**a**) CEM100_MK0, (**b**) CEM50_MK 50.

The fresh state properties were quantified to confirm that the CEM-MK mortars remained highly workable under the fixed water/binder ratio and constant superplasticiser amount adopted in the experimental program. Flowability was measured using the flow table spread test [56], and the resulting spread diameters for the CEM-MK series are reported in Table 3 and selectively in Figure 4.

The measured flow diameter decreased monotonically from 280 mm for the CEM control to 240 mm at 50% MK, with intermediate values of 270 mm (10% MK), 260 mm (20% MK), 255 mm (30% MK), and 250 mm (40% MK). The gradual loss of spread with increasing MK replacement is consistent with the physical characteristics of MK. MK typically exhibits a high specific surface area and angular particle morphology relative to cement, which increases water demand and intensifies interparticle friction, thereby raising yield stress and reducing the free flow [69]. Despite the observed reduction in spread, the CEM-MK mortars remained within a relatively high level of workability window defined by the experimental criterion considered here, i.e., a reference spread in the 250–300 mm range and stable placement without external vibration. The progressive reduction in spread shown in Figure 4 indicates that MK increased mixture cohesiveness rather than inducing instability; this behaviour is consistent with the literature [28].

The results indicate that at up to 20% MK, the spread decreased moderately (280 mm to 260 mm), indicating that workable behaviour can be maintained with limited flow loss. Beyond 30% MK, the spread approached the lower bound recorded for the CEM-MK series (255 mm to 240 mm from 30% to 50% MK), implying that further MK addition increasingly dominates the rheological response, and that mix redesign would be required if high MK contents are targeted [19,69].

### 3.2. Mechanical and Transport Properties

#### 3.2.1. Compressive Strength

**Figure 5:**
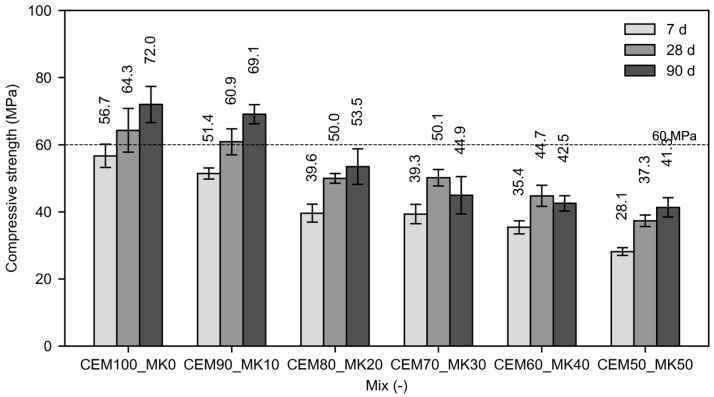
Compressive strength development of CEM-MK mortars at 7, 28 and 90 days.

The development of compressive strength of the CEM-MK mortars is shown in Figure 5 and Table 4, which present the results obtained at curing ages of 7, 28 and 90 days as a function of the MK replacement level. In most binder systems, compressive strength increases with curing age, while increasing replacement of cement by MK leads to a gradual reduction in strength. The control mix (CEM100_MK0) increases from 56.7 MPa at 7 days to 64.3 MPa at 28 days and 72.0 MPa at 90 days. A 10% MK replacement leads to strengths close to the control (CEM90_MK10, 51.4, 60.9 and 69.1 MPa at 7, 28 and 90 days, respectively). Higher replacement levels result in progressively lower strengths at all ages. CEM80_MK-20 reaches 39.6, 50.0 and 53.5 MPa, while CEM50_MK50 reaches 28.1, 37.3 and 41.1 MPa at 7, 28 and 90 days. Strengths gained between 7 and 28 days are broadly similar across mixtures (approximately 8–11 MPa), whereas changes from 28 to 90 days are smaller or, in some cases, negative on average. For CEM70_MK30 and CEM60_MK40, the trends seem to be slightly negative, yet generally with a higher standard deviation that may offset the perceived trends.

The influence of MK on compressive strength reflects the interaction of physical and chemical mechanisms. At early ages, the fine particle size of MK can enhance particle packing and provide nucleation sites, thereby modifying hydration kinetics [7,70]. With continued curing, MK reacts pozzolanically with portlandite to form additional binding phases, including secondary C–S–H and Al-bearing hydrates, which refine pore structure and densify the interfacial transition zone, potentially supporting later-age strength development [71]. Conversely, MK may retard hydration in some systems, attributed to early precipitation of Al-rich C–A–S–H and sulphate–aluminate interactions that alter C–S–H nucleation and growth [72]. At high replacement levels, the relatively high specific surface area of MK increases water demand, leading to strength reductions [27,71].

The literature generally identifies an optimal MK replacement in the range of 10–20%, depending on mixture design, curing conditions, admixture use and MK quality [28]. Ref. [71] reported a peak 28-day strength at 15% MK with a slight reduction at 20%, while Ref. [27] found 10% MK to be more favourable than 20%, noting the associated increase in water demand. With appropriate workability control, 20% MK can lead to improved later-age strength [22,73]. In contrast, the higher replacement levels examined in Table 4 (up to 50%) exhibit an almost monotonic reduction in strength with increasing MK content, indicating that dilution effects and/or uncompensated water demand dominate.

#### 3.2.2. Flexural Strength

**Figure 6:**
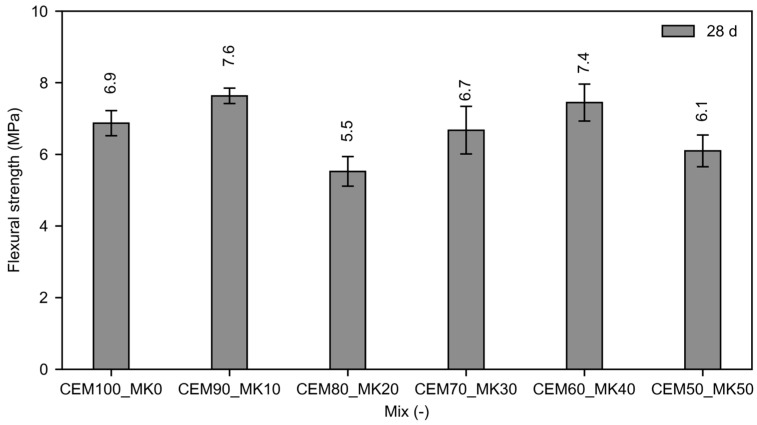
Flexural strength of CEM-MK mortars at 28 days.

The flexural strength results of the CEM-MK mortars at 28 days are presented in Figure 6, which shows the variation in flexural strength as a function of MK replacement level. Flexural strength was evaluated at a single curing age, as it is commonly considered a complementary mechanical indicator reflecting tensile performance and crack-bridging capacity rather than long-term hydration kinetics. The figure shows that partial replacement of CEM with MK generally maintains or enhances 28-day flexural strength up to about 40% MK, with the highest values observed for the 90CEM_10MK and 60CEM_40MK mixes. A reduction in flexural strength occurs at higher replacement levels (≥50% MK), which may indicate a threshold beyond which dilution of clinker and changes in binder packing and hydration products outweigh the pozzolanic benefits of MK.

The flexural strength results shown in Figure 6 confirm that moderate MK replacement levels (10–40%) can preserve or slightly enhance the tensile-related mechanical performance of CEM-based mortars, while higher replacement levels lead to increased brittleness and reduced bending resistance. The unclear trends for flexural strength are due to sample inhomogeneity, such as random distribution of pores, and inherent brittleness of the material in flexure and tension. Flexural strength was therefore retained at 28 days, consistent with common mortar strength assessment practice under BS EN 1015-11 [57] and interpreted as a complementary indicator of tensile-related behaviour rather than the primary metric of hydration development, which is captured by compressive testing. This is supported by previous studies showing that tensile-related properties in MK-modified systems generally follow the same trends as compressive strength, including portlandite consumption, pore refinement, reduced microcracking and formation of additional C–S–H/C–A–S–H products [10,22,30].

#### 3.2.3. Capillary Absorption

**Figure 7:**
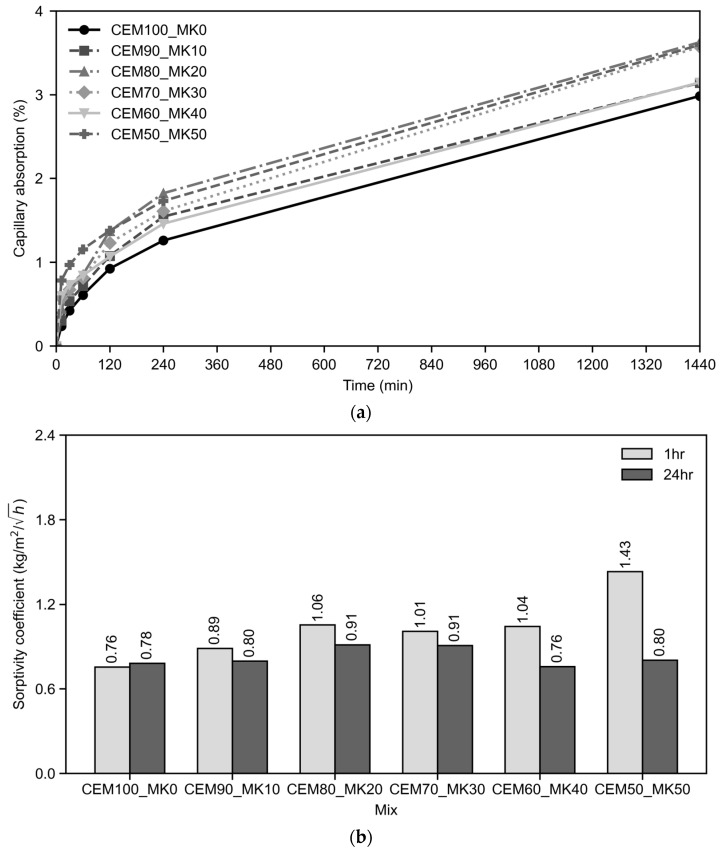
Capillary water absorption results for CEM-MK mixes: (**a**) capillary absorption by weight (%) versus time, (**b**) sorptivity coefficients.

Figure 7a shows the capillary uptake for the tested mixes with respect to time, based on measurements at 12 min, 30 min, 1 h, 2 h, 4 h and 24 h. It is shown that the CEM100_MK0 mix, shown in a continuous black curve and black dot markers, acting as a baseline mix, has the lowest uptake with respect to blended mixes. For the initial absorption, considered here for a time interval of up to 1 h, the CEM50_MK50 mix has the highest capillary uptake. This mix is shown in a grey dash–dot curve and cross markers. The intermediate mixes are between the two extremes. Capillary uptake is known to be sensitive to total porosity, pore continuity and effective pore radius.

The sorptivity coefficients shown in Figure 7b provide a clearer quantitative interpretation of the capillary absorption results by distinguishing between the initial uptake stage (0–1 h) and the subsequent transport stage (1–24 h). The initial sorptivity coefficient increased from 0.76 kg/m^2^/h^0.5^ for the control mix to 0.89, 1.06, 1.01, 1.04 and 1.43 kg/m^2^/h^0.5^ for 10, 20, 30, 40 and 50% MK, respectively, indicating that MK reduces early absorption gradually but not significantly. The secondary sorptivity coefficient (1–24 h) varied within a narrower range, from 0.78 to 0.91 kg/m^2^/h^0.5^, with values of 0.80, 0.91, 0.91, 0.76 and 0.80 kg/m^2^/h^0.5^ for 10, 20, 30, 40 and 50% MK, compared with 0.78 kg/m^2^/h^0.5^ for the control. This indicates that the secondary-to-initial sorptivity ratio decreased from about 1.03 in the control to 0.90, 0.86, 0.90, 0.75, and 0.56, respectively, showing that mixes with higher MK content absorbed water rapidly initially, but the secondary and subsequent rates of capillary transport reduced.

This behaviour suggests rapid filling of accessible near-surface pores followed by slower, longer-range transport through a more tortuous or partially disrupted pore network. This is aligned with the microstructural assessments described in the subsequent section, which, in short, indicate that moderate MK contents promoted portlandite consumption and secondary C-A-S-H formation, which are expected to refine pore structure, whereas higher MK contents showed unreacted MK and greater microcracking at 50% MK. Similar mechanisms have been reported in the literature, where MK at moderate contents reduced sorptivity, porosity and permeability through pore refinement and reduced pore connectivity [17,22,23], while MK-containing systems have also been shown to reduce larger capillary pores and increase tortuosity or lower critical pore size [16,34]. For high MK mixes, however, the fixed-mixture design with respect to water-to-binder and admixture content appears to have preserved or amplified early absorption and stabilised beyond one hour of absorption.

### 3.3. Microstructural Analysis Results

#### 3.3.1. SEM/EDS

MK is mainly composed of two principal oxides, SiO_2_ and Al_2_O_3_, which represent approximately 50% and 40%, respectively, of its overall chemical composition [47,74,75]. Its primary effect within the concrete matrix is the reduction of Ca(OH)_2_ during the hydration process, which promotes the formation of secondary hydration products such as the C-S-H gel [76]. This crystalline phase is responsible for the bonding behaviour of the cement paste and directly influences concrete durability, porosity, and chloride ion diffusivity [27]. Additionally, MK enhances the mechanical performance of cement-based pastes, mortars, and concretes by reducing Ca(OH)_2_ content, refining pore size distribution, and improving paste–aggregate bonding [77]. C-A-S-H is formed through the interaction of cement hydration products with the reactive SiO_2_ and Al_2_O_3_ phases supplied by MK [78]. The incorporation of aluminium into the silicate structure modifies the chemistry of the binding gel, leading to the development of C-A-S-H. In SEM micrographs, this phase typically appears slightly darker than conventional C-S-H [79].

**Figure 8:**
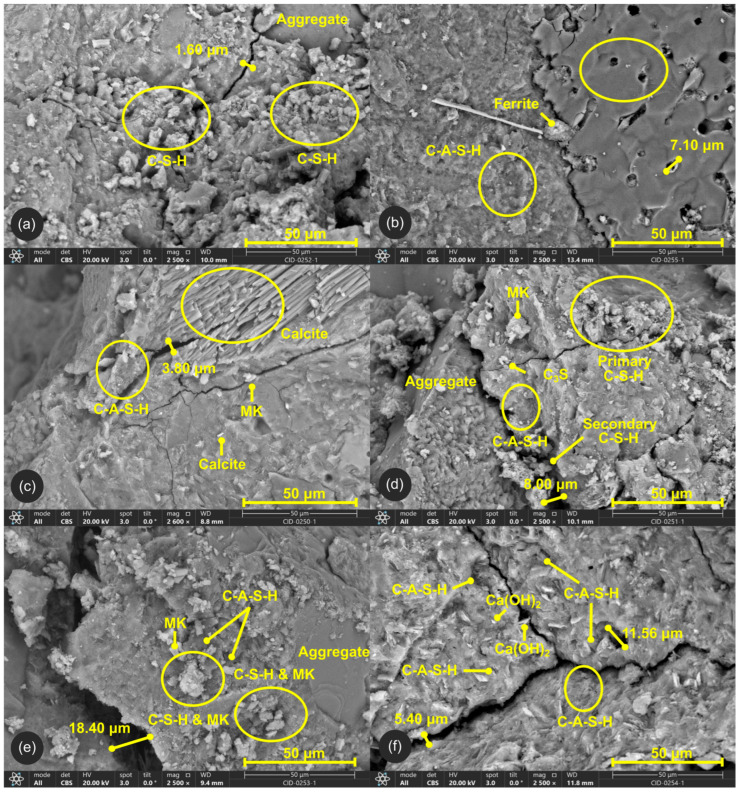
SEM images for (**a**) control, (**b**) 10%, (**c**) 20%, (**d**) 30%, (**e**) 40%, and (**f**) 50% replacement samples.

**Figure 9:**
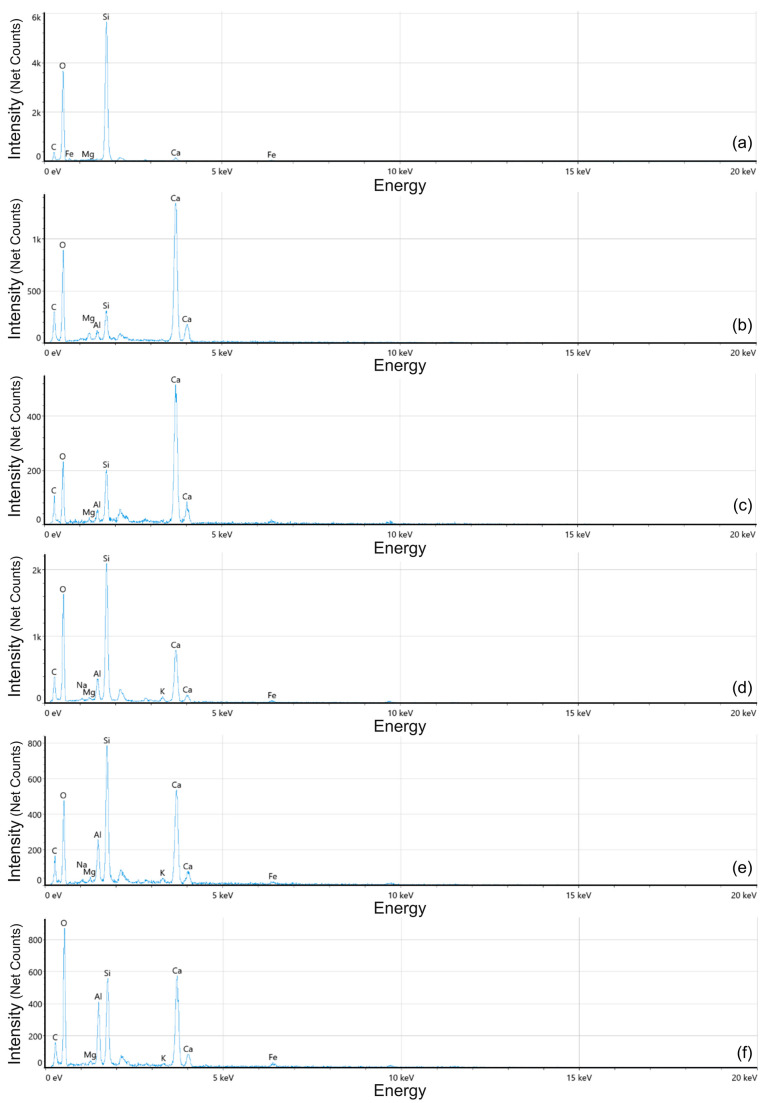
EDS images for (**a**) control, (**b**) 10%, (**c**) 20%, (**d**) 30%, (**e**) 40%, and (**f**) 50% replacement samples.

The SEM/EDS results are shown in Figure 8 and Figure 9. The control sample (CEM100_MK0), with no MK replacement, is characterised by a dominant formation of conventional C-S-H gel coating on the fine aggregates. However, microcracks up to 1.6 µm in width were observed. In the 10% replacement mixture (CEM90_MK10), the microstructure shows a denser formation of calcium carbonate (CaCO_3_), with embedded voids measuring approximately 4.90 and 7.10 µm.

Some ferrite phases were also identified between the C-A-S-H and CaCO_3_ regions. The 20% replacement sample (CEM80_MK20) exhibited a high presence of C-A-S-H. Unreacted MK particles were also observed, indicating incomplete participation in the pozzolanic reaction. CaCO_3_ nucleation was present throughout the matrix, and microcrack openings increased up to 3.80 µm. The sample with 30% MK replacement (CEM70_MK30) contained both primary and secondary C-S-H gels, alongside impure C_3_S remnants. Primary C-S-H is typically formed directly from cement hydration, whereas secondary C-S-H results from subsequent pozzolanic reactions and is commonly located within pores and along microcrack surfaces, as shown in Figure 8d. In this mixture, microcracks became more pronounced, with widths of up to 8 µm. At 40% replacement (CEM60_MK40), a substantial amount of unreacted MK was detected. Some crystalline structures showed secondary C-S-H forming around the unreacted MK particles. Microcracks increased significantly, reaching widths of up to 18 µm. Finally, the 50% replacement sample (CEM50_MK50) displayed slight changes in microstructure, including the presence of Ca(OH)_2_, indicating incomplete consumption of portlandite during hydration. Larger cracks up to 5.40 µm were observed throughout the matrix, accompanied by voids measuring approximately 11.56 µm.

SEM/EDS analysis indicated that the formation of C-A-S-H is strongly dependent on the incorporation of MK in the mixture. The addition of MK to CEM I modifies both the structure and chemical composition of calcium silicate hydrate, particularly by increasing the Al/Si ratio. In this context, the detection of Al can be considered an indicator of C-A-S-H formation within the analysed samples. In the control mixture, the presence of Al was negligible. As shown in Figure 9a, the corresponding EDS spectrum did not exhibit a significant Al peak. In contrast, mixtures containing MK displayed a progressive increase in Al content as the replacement level increased. This trend was especially evident at 50% MK substitution, where the EDS spectrum showed a pronounced Al peak intensity. These findings are consistent with previous studies reporting that MK-blended systems exhibit higher Al_2_O_3_ contents due to the incorporation of Al into the C-A-S-H structure and the formation of alumina-rich hydration products [6,27].

#### 3.3.2. TGA

**Figure 10:**
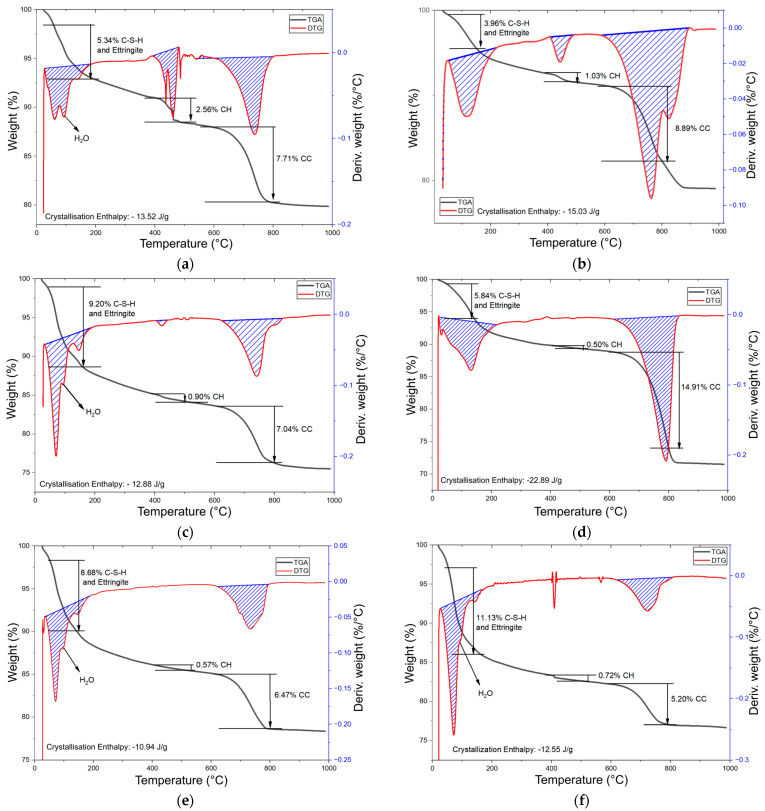
TGA for (**a**) control, (**b**) 10%, (**c**) 20%, (**d**) 30%, (**e**) 40%, and (**f**) 50% replacement samples.

The thermogravimetric curves presented in Figure 10 exhibit three distinctive mass-loss regions that correspond to well-established decomposition events in cementitious systems: (i) 50–150 °C, associated with the dehydration of C-S-H gel, ettringite, and evaporation of free water; (ii) 400–500 °C, related to the dehydroxylation of portlandite (CH); and (iii) 600–800 °C, linked to the decarbonation of calcium carbonate (CC) [79,80]. The control mixture shows the highest portlandite content, exhibiting a mass loss of 2.56% in the 400–500 °C interval. Without MK pozzolanic activity, portlandite is not consumed by secondary reactions, resulting in the most pronounced CH signal among all mixtures.

The MK-containing mixtures exhibit the classical signature of pozzolanic activity. At low to moderate replacement levels (10–30%), the mass loss associated with portlandite decreases markedly compared to the control mixture, confirming CH consumption and the formation of secondary hydration products. This reduction reaches its lowest value at 30% MK replacement, where the CH-related mass loss drops to only 0.50%, approximately five times lower than the control. At this same replacement level, CC reaches its maximum value (14.91%), which is attributed to carbonation processes occurring in the sample. This behaviour is consistent with SEM observations showing that mixtures containing 20–30% MK develop highly compacted microstructures dominated by secondary C-S-H gels formed through the pozzolanic reaction between MK and available portlandite.

However, mixtures with higher MK replacement levels (40–50%) deviate from this optimal performance. In these samples, the TGA curves show a less pronounced reduction in CH-associated mass loss. This trend corresponds with SEM evidence revealing the presence of unreacted MK at 40–50% replacement and the detection of Ca(OH)_2_ in the 50% MK mixture. These findings indicate that excessive MK leads to insufficient calcium availability or incomplete activation, causing part of the MK to remain unreacted and limiting the extent of the pozzolanic reaction.

One of the main properties affected by the incorporation of MK was compressive strength. The development of compressive strength is closely associated with the proper formation and nucleation of C-S-H resulting from C_3_S hydration, as well as with the availability of CH to sustain the pozzolanic reaction [8,23]. However, high MK contents significantly consume CH, which affects the balance between primary hydration and secondary pozzolanic reactions [19,20]. As shown in Figure 10, increasing MK replacement levels led to a considerable reduction in CH content. Furthermore, as observed in Figure 8d, high MK incorporation appeared to hinder complete C_3_S hydration, as evidenced by the presence of unreacted C_3_S particles within the matrix. These microstructural findings are consistent with the compressive strength results, where increasing MK replacement levels resulted in a progressive reduction in strength.

#### 3.3.3. XRD

X-ray Diffraction (XRD) analysis was performed to complement the SEM and thermogravimetric (TGA) results by identifying crystalline phases and assessing the amorphous character of the raw materials used in this study. Since the main binding phases in hydrated cementitious systems, particularly C-S-H and C-A-S-H, are poorly crystalline or largely X-ray amorphous, XRD was primarily employed to evaluate phase assemblage, amorphous content and portlandite-related trends rather than to directly quantify hydration products [80,81].

**Figure 11:**
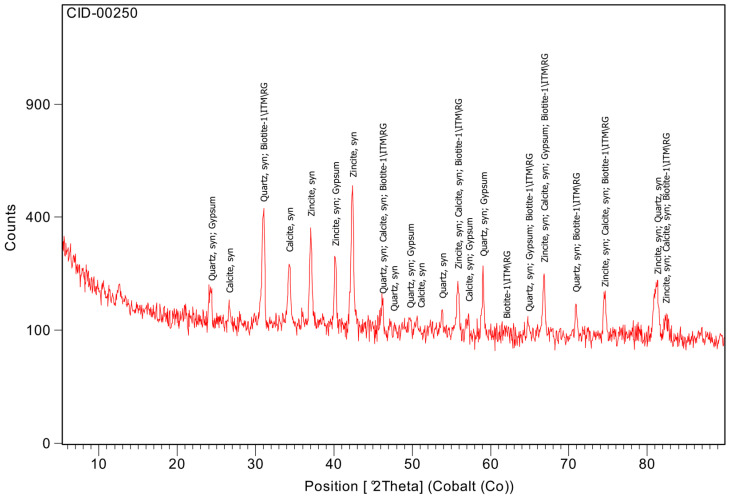
XRD of CEM I.

The diffraction pattern of the CEM I (CID-00250 Figure 11) indicated a predominantly amorphous character (76.5%) together with crystalline quartz (11.2%), calcite (9.4%), biotite (1.6%) and gypsum (1.3%). The presence of gypsum corresponds to the sulphate source used to regulate setting, while calcite is likely associated with limestone addition or minor carbonation during storage. The high amorphous fraction reflects the reactive clinker phases and poorly crystalline constituents responsible for Ca(OH)_2_ generation during hydration.

**Figure 12:**
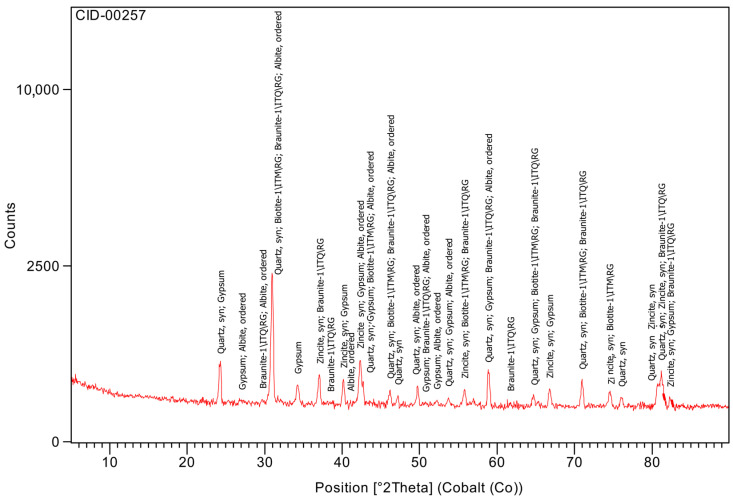
XRD of sand.

The sand (CID-00257, Figure 12) exhibited a predominantly siliceous composition, with quartz representing 54.5% of the crystalline phases and an amorphous fraction of 39.9%. Minor phases such as gypsum, biotite, braunite and albite were also detected. As expected, the aggregate does not participate chemically in hydration reactions but contributes to the overall crystalline background of blended systems.

**Figure 13:**
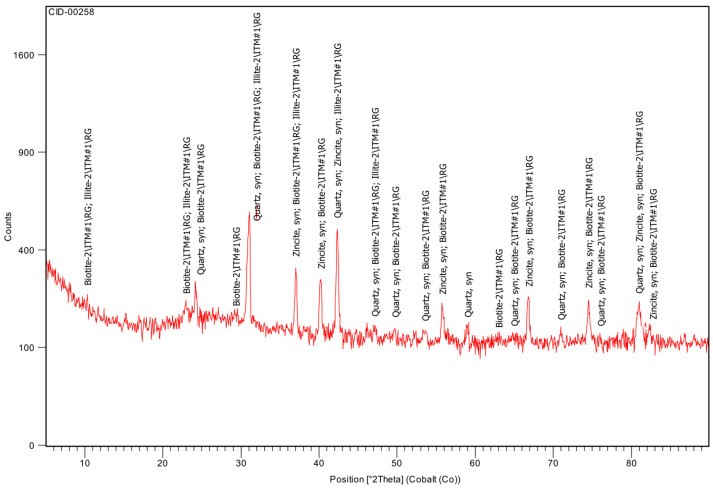
XRD of MK.

The MK (CID-00258, Figure 13) presented a significant amorphous fraction of 42.5%, characterised by a broad diffuse halo between approximately 15° and 35° 2θ (Co radiation). This diffuse hump is characteristic of calcined kaolinitic clays and reflects the formation of a disordered aluminosilicate structure after dehydroxylation of kaolinite. Crystalline illite (24.2%), quartz (18.1%) and biotite (15.2%) were identified as residual mineral phases. The predominance of the amorphous aluminosilicate phase confirms the high pozzolanic potential of the MK, consistent with previous studies demonstrating that calcined clays derive their reactivity from this disordered SiO_2_-Al_2_O_3_ system [7].

Although hydration products such as C-S-H and C-A-S-H are not directly detectable by XRD due to their largely amorphous nature [80], the mineralogical composition of the raw materials provides the chemical basis for the observed mechanical behaviour. During cement hydration, Ca(OH)_2_ released from clinker reactions reacts with the reactive aluminosilicate phases of MK, forming additional C-A-S-H-type gels. At moderate replacement levels, this secondary pozzolanic reaction promotes matrix densification and pore refinement, which explains the relatively limited reduction in compressive strength compared to the control mixture [82].

At higher replacement levels, however, the availability of Ca(OH)_2_ may become insufficient to fully react with the increased MK content, leading to a calcium-limited system. In such cases, part of the MK remains unreacted, and the formation of strength-contributing secondary hydrates is reduced. This limitation of MK reactivity at high replacement ratios has been experimentally demonstrated and quantified by [7], who showed that incomplete reaction of calcined clay is associated with insufficient Ca(OH)_2_ availability. The persistence of residual aluminosilicate phases therefore corroborates the existence of an optimal replacement range beyond which mechanical performance declines due to combined dilution and kinetic constraints.

The absence of clearly distinguishable calcite peaks in blended hydrated systems may be attributed to limited carbonation under water-curing conditions, peak overlap with the broad amorphous hump associated with C-A-S-H, or the formation of poorly crystalline carbonates below the XRD detection threshold. Similarly, although ettringite formation was inferred from characteristic dehydration regions in TGA, it was not clearly resolved in XRD patterns. This discrepancy can be explained by its relatively low abundance, limited crystallinity and possible partial transformation into AFm-type phases during hydration. As discussed in the cement chemistry literature [65,81], TGA is more sensitive to chemically bound water and can detect hydration-related mass losses even when crystalline signatures are weak or masked in diffraction patterns.

Therefore, the XRD results confirm the highly amorphous and reactive nature of the MK and provide mineralogical support to the thermogravimetric and mechanical findings. The evolution of the binder system is therefore chemically governed by the balance between calcium availability from clinker hydration and the reactivity of the amorphous aluminosilicate phase, which ultimately controls the formation of secondary hydrates and the resulting mechanical performance.

### 3.4. Carbon Footprint Analysis

In the present study, the carbon footprint implications of MK incorporation were evaluated through a comparative, binder-based assessment, focusing on the reduction in CEM content associated with increasing MK replacement levels. Based on the mix proportions (Table 2) and the emission factors reported in Table 5, the calculated embodied carbon values per cubic metre were 541.0 kgCO_2_e/m^3^ for CEM100_MK0, decreasing progressively to 506.1 kgCO_2_e/m^3^ (CEM90_MK10), 467.0 kgCO_2_e/m^3^ (CEM80_MK20), 428.4 kgCO_2_e/m^3^ (CEM70_MK30), 389.0 kgCO_2_e/m^3^ (CEM60_MK40), and 350.9 kgCO_2_e/m^3^ (CEM50_MK50). At low to moderate replacement levels (10–30%), the reduction in CEM content leads to a proportional decrease in CO_2_ emissions associated with binder production, while maintaining or enhancing mechanical and durability performance. Within this range, the carbon efficiency of the mortar system is maximised, as reductions in embodied CO_2_ are achieved without significantly compromising the compressive strength, flexural strength, or transport-related durability indicators.

At higher MK replacement levels (40–50%), although the nominal clinker content is further reduced, the environmental benefits are partially offset by the deterioration in mechanical and durability performance observed experimentally. From a functional perspective, such mixtures may require increased binder content, which can negate the apparent carbon savings associated with higher cement substitution. This observation reinforces the concept that clinker replacement alone is not sufficient to guarantee meaningful carbon reduction if material performance is compromised [35].

It is also acknowledged that the carbon footprint of MK itself depends on calcination temperature, clay source, and processing efficiency. Nevertheless, comprehensive assessments consistently show that, even when accounting for calcination-related emissions, MK exhibits a substantially lower embodied CO_2_ intensity than clinker [3]. Consequently, partial substitution of CEM with MK remains an effective decarbonisation strategy when applied within a replacement range that ensures efficient utilisation of the material’s pozzolanic reactivity. Therefore, the combined mechanical, durability, microstructural, and environmental results indicate that MK replacement levels in the range of approximately 10–30% provide the most favourable balance between performance and sustainability.

### 3.5. Discussion

The results show that the response of high-workability CEM-MK mortars is governed by a balance between the beneficial filler–pozzolanic action of MK at low-to-moderate replacement and dilution- or calcium-limited behaviour at higher replacement levels. Fresh property testing showed a gradual reduction in spread from 275 mm for CEM100_MK0 to 245 mm for CEM50_MK50, indicating that the inferred particle size and morphology of MK are responsible for increased water demand and internal friction [10,13,29]. Importantly, however, workability remained acceptable for all investigated mixes, with a reduction of only 10% at 50% MK content.

Compressive strength tests indicated that 10% MK replacement gave similar values to the control, with values showing less than 10% reduction in properties at all ages. Beyond this level, strength decreased progressively, with reductions of up to 50% relative to the control for the 50% MK mix. This behaviour is consistent with the wider MK literature, where optimum compressive strength is commonly reported at around 5–20% replacement, depending on the binder system and mixture design [10,21,22]. The flexural results broadly support the same interpretation, although with greater scatter, which is expected for a brittle property that is more sensitive to local defects and pore distribution. Flexural strength was maintained or slightly improved up to 40% MK, while a reduction was observed at 50% MK. The divergence between compressive and flexural trends at some intermediate replacement levels may therefore reflect local heterogeneity and crack sensitivity rather than a fundamentally different hydration mechanism.

The capillary absorption results indicate that the control mix showed the lowest overall uptake. The sorptivity coefficients show that the initial sorptivity increased with MK content, whereas the secondary sorptivity varied over a narrower range, and the secondary-to-initial sorptivity ratio decreased gradually. This suggests that mixes with high MK contents absorbed water rapidly at early times through accessible near-surface pores, but that subsequent transport became less efficient, consistent with increased tortuosity or partial disruption of continuous capillary pathways [16,17,34].

The SEM/EDS, TGA and XRD results support further understanding of the mechanical and transport behaviour observed. SEM showed that the 20–30% MK mixes contained abundant C-A-S-H and secondary C-S-H, whereas 40–50% MK mixes contained substantial unreacted MK, larger cracks and visible voids. TGA confirmed this trend, with CH-related mass loss being highest in the control mix, decreasing visibly in the 10–30% MK mixtures, and reaching a minimum at 30% MK. These trends demonstrate active pozzolanic consumption of portlandite and formation of secondary hydrates. At 40–50% MK, the CH reduction became less pronounced and residual Ca(OH)_2_ was visible in the SEM micrographs, possibly indicating incomplete reaction and insufficient calcium availability. XRD further confirmed that the MK used here was highly amorphous and reactive, but also supported the interpretation that high replacement levels lead to a calcium-limited system in which part of the aluminosilicate phase remains unreacted. This integrated interpretation agrees with the existing literature, which shows that MK promotes secondary hydrates, pore refinement and matrix densification at moderate contents, but that the reaction becomes constrained when available calcium is insufficient [11,14,16,33].

## 4. Conclusions

This research study evaluated the effect of metakaolin (MK) as a partial replacement of cement (CEM I) in high-workability mortars, considering fresh-state behaviour, mechanical performance, durability-related transport properties, microstructural evolution, and carbon footprint implications. Based on the experimental results, the following conclusions are drawn:

- All mixtures remained within the targeted high-workability range under a constant water-to-binder ratio and fixed superplasticiser amount. Increasing MK content reduced flowability due to its high fineness; however, high workability was maintained for replacement levels up to 20%, demonstrating the feasibility of incorporating moderate MK contents without compromising workability.
- Compressive strength increases with curing age for all mixes, but increasing MK content led to a gradual strength reduction, with 10% MK retaining ~90–95% of the control strength at all ages. Higher MK replacements result in monotonic losses, with 20% and 50% MK achieving approximately 75–80% and 55–60% of the 28-day control strength, respectively.
- At 28 days, partial replacement of cement with MK up to about 40% maintains or, in some cases, improves flexural strength, with peak performance observed for the CEM 90_MK10 and CEM60_MK40 mixes, while higher replacement levels (≥50% MK) lead to a clear reduction in strength, indicating a practical upper limit to beneficial MK incorporation in these mortars.
- Capillary absorption developed differently in the two uptake stages. In the first stage (up to 1 h), the sorptivity coefficient increased relative to the control by about 17% for 10% MK, 39% for 20% MK, 33% for 30% MK, 37% for 40% MK, and 88% for 50% MK. In the second stage (1–24 h), the sorptivity coefficient remained much closer to the control, with −3–17% for all replacement levels, showing that the influence of MK was much more pronounced during the initial uptake period than during the later transport stage.
- SEM, TGA, and XRD analyses confirmed that moderate MK replacement promotes portlandite consumption and the formation of dense C-A-S-H-type hydration products, leading to a more homogeneous microstructure for up to 20–30% replacements. Excessive MK contents resulted in unreacted MK, increased microcracking, and incomplete pozzolanic reaction, explaining the observed performance losses.
- Replacing CEM with MK led to proportional reductions in embodied CO_2_ emissions. When functional performance was considered, MK replacement levels in the range of 20% provided the most efficient balance between mechanical performance, durability, and carbon reduction, whereas higher replacement levels showed diminishing practical benefits.

## Figures and Tables

**Table 1:** Mix proportions for all CEM-MK mortars per cubic metre.

Mix	CEM I 52.5 (kg)	MK (kg)	Fine Aggregates (kg)	Superplasticiser (kg)	Water (kg)	Water/Binder (w/b)	Fine Aggregates/Binder
CEM100_MK0	592	0	1496	8.8	360	0.6	2.5
CEM90_MK10	535	59	1496	8.8	360	0.6	2.5
CEM80_MK20	473	118	1496	8.8	360	0.6	2.5
CEM70_MK30	412	176	1496	8.8	360	0.6	2.5
CEM60_MK40	350	234	1496	8.8	360	0.6	2.5
CEM50_MK50	290	290	1496	8.8	360	0.6	2.5

**Table 2:** CO_2_ emission factors for all constituent materials used in the CEM-MK mix designs.

Material	Emission Factor (kg CO_2_/kg) [38]
CEM I 52.5	0.86
MK (MK—Argical 1000)	0.24
Fine aggregate (river sand)	0.008
Superplasticiser (PCE)	2.25
Water	0.0003

**Table 3:** Flow table results for all CEM-MK mixes.

Mix ID	Flow Diameter (mm)
CEM100_MK0	275
CEM90_MK10	268
CEM80_MK20	260
CEM70_MK30	255
CEM60_MK40	250
CEM50_MK50	245

**Table 4:** Mechanical properties.

MIX ID	Compressive Strength (MPa)			Flexural Strength (MPa)
-	7 Days	28 Days	90 Days	28 Days
CEM100_MK0	56.7 ± 3.8	64.3 ± 6.5	72.0 ± 5.4	6.87 ± 0.35
CEM90_MK10	51.4 ± 1.7	60.9 ± 7.9	69.1 ± 2.9	7.63 ± 0.22
CEM80_MK20	39.6 ± 2.7	50.0 ± 1.5	53.5 ± 5.3	5.52 ± 0.42
CEM70_MK30	39.3 ± 2.8	50.1 ± 2.4	44.9 ± 5.6	6.67 ± 0.67
CEM60_MK40	35.4 ± 1.9	44.7 ± 3.1	42.5 ± 2.3	7.45 ± 0.52
CEM50_MK50	28.1 ± 1.2	37.3 ± 1.7	41.3 ± 2.8	6.10 ± 0.45

**Table 5:** Embodied carbon results for all CEM-MK mixes.

Mix ID	Embodied Carbon A1–A3 (kgCO_2_e/m^3^)
CEM100_MK0	541.0
CEM90_MK10	506.1
CEM80_MK20	467.0
CEM70_MK30	428.4
CEM60_MK40	389.0
CEM50_MK50	350.9

## Data Availability

The original contributions presented in this study are included in the article. Further inquiries can be directed to the corresponding authors.

## References

[1] Andrew R.M. (2018). Global CO_2_ emissions from cement production. Earth Syst. Sci. Data.

[2] Scrivener K.L., Martirena F., Bishnoi S., Maity S. (2018). Calcined clay limestone cements (LC3). Cem. Concr. Res..

[3] Habert G., Miller S.A., John V.M., Provis J.L., Favier A., Horvath A., Scrivener K.L. (2020). Environmental impacts and decarbonization strategies in the cement and concrete industries. Nat. Rev. Earth Environ..

[4] Juenger M.C.G., Snellings R., Bernal S.A. (2019). Supplementary cementitious materials: New sources, characterization, and performance insights. Cem. Concr. Res..

[5] Zhang C., Bompa D.V., Biswal S., Wang Y. Performance of one-part alkali-activated materials incorporating fly ash and slag. Proceedings of the 15th fib International PhD Symposium.

[6] Khan M.I., Khan H.U., Azizli K., Sufian S., Man Z., Siyal A.A., Muhammad N., Rehman M.F. (2017). The pyrolysis kinetics of the conversion of Malaysian kaolin to metakaolin. Appl. Clay Sci..

[7] Avet F., Li X., Scrivener K. (2018). Determination of the amount of reacted metakaolin in calcined clay blends. Cem. Concr. Res..

[8] Williams A., Markandeya A., Stetsko Y., Riding K.A., Zayed A. (2016). Cracking potential and temperature sensitivity of metakaolin concrete. Constr. Build. Mater..

[9] Li M., Zheng K., Chen L., Prateek G., Zhou X., Yuan Q. (2024). Using metakaolin to improve properties of aged Portland cement: Effectiveness and the mechanism. Constr. Build. Mater..

[10] Kavitha O.R., Shanthi V.M., Arulraj G.P., Sivakumar P. (2015). Fresh, micro- and macrolevel studies of metakaolin blended self-compacting concrete. Appl. Clay Sci..

[11] Amer A.A., El-Hoseny S. (2017). Properties and performance of metakaolin pozzolanic cement pastes. J. Therm. Anal. Calorim..

[12] Zhao D., Khoshnazar R. (2020). Microstructure of cement paste incorporating high volume of low-grade metakaolin. Cem. Concr. Compos..

[13] Sujjavanich S., Suwanvitaya P., Chaysuwan D., Heness G. (2017). Synergistic effect of metakaolin and fly ash on properties of concrete. Constr. Build. Mater..

[14] Shah V., Parashar A., Scott A. (2022). Understanding the importance of carbonates on the performance of Portland metakaolin cement. Constr. Build. Mater..

[15] Li W., Hua L., Shi Y., Wang P., Liu Z., Cui D., Sun X. (2022). Influence of metakaolin on the hydration and microstructure evolution of cement paste during the early stage. Appl. Clay Sci..

[16] Cai R., Tian Z., Ye H., He Z., Tang S. (2021). The role of metakaolin in pore structure evolution of Portland cement pastes revealed by an impedance approach. Cem. Concr. Compos..

[17] Bakera A.T., Alexander M.G. (2019). Use of metakaolin as supplementary cementitious material in concrete, with focus on durability properties. RILEM Tech. Lett..

[18] Papp V., Ardelean I., Bulátkó A., László K., Csík A., Janovics R., Kéri M. (2025). Effect of metakaolin and fly ash on the early hydration and pore structure of Portland cement. Cem. Concr. Res..

[19] Rashad A.M. (2013). Metakaolin as cementitious material: History, scours, production and composition—A comprehensive overview. Constr. Build. Mater..

[20] Kannan V., Ganesan K. (2014). Mechanical properties of self-compacting concrete with binary and ternary cementitious blends of metakaolin and fly ash. J. S. Afr. Inst. Civ. Eng..

[21] Elavarasan S., Priya A.K., Ajai N., Akash S., Annie T.J., Bhuvana G. (2021). Experimental study on partial replacement of cement by metakaolin and GGBS. Mater. Today Proc..

[22] Al-Hashem M.N., Amin M.N., Ajwad A., Afzal M., Khan K., Faraz M.I., Qadir M.G., Khan H. (2022). Mechanical and durability evaluation of metakaolin as cement replacement material in concrete. Materials.

[23] Badogiannis E., Kakali G., Dimopoulou G., Chaniotakis E., Tsivilis S. (2005). Metakaolin as a Main Cement Constituent. Exploitation of Poor Greek Kaolins. Cem. Concr. Compos..

[24] Geu M.J., Zhuge Y., Ma X., Pham T.M. (2026). Systematic review of physical, mechanical and durability performances of metakaolin concrete. Appl. Clay Sci..

[25] Dong Y., Pei L., Fu J., Yang Y., Liu T., Liang H., Yang H. (2022). Investigating the mechanical properties and durability of metakaolin-incorporated mortar by different curing methods. Materials.

[26] Dhakal M., Scott A.N., Shah V., Dhakal R.P., Clucas D. (2021). Development of a MgO-metakaolin binder system. Constr. Build. Mater..

[27] Wild S., Khatb J.M., Jones A. (1996). Relative Strength. Pozzolanic activity and cement hydration in superplasticised metakaolin concrete. Cem. Concr. Res..

[28] Siddique R., Klaus J. (2009). Influence of metakaolin on the properties of mortar and concrete. Appl. Clay Sci..

[29] Asghari Y., Mohammadyan-Yasouj S.E., Rahimian Koloor S.S. (2023). Utilization of metakaolin on the properties of self-consolidating concrete: A review. Constr. Build. Mater..

[30] Ismail M.K., Hassan A.A.A. (2016). Use of metakaolin on enhancing the mechanical properties of self-consolidating concrete containing high percentages of crumb rubber. J. Clean. Prod..

[31] Qin Z., Ma C., Zheng Z., Long G., Chen B. (2020). Effects of metakaolin on properties and microstructure of magnesium phosphate cement. Constr. Build. Mater..

[32] Dhandapani Y., Sakthivel T., Santhanam M., Gettu R., Pillai R.G. (2018). Mechanical properties and durability perfor-mance of concretes with limestone calcined clay cement (LC3). Cem. Concr. Res..

[33] Zunino F., Scrivener K. (2021). The reaction between metakaolin and limestone and its effect in porosity refinement and mechanical properties. Cem. Concr. Res..

[34] Tang J., Wei S., Li W., Ma S., Ji P., Shen X. (2019). Synergistic effect of metakaolin and limestone on the hydration properties of Portland cement. Constr. Build. Mater..

[35] Thorne J., Bompa D.V., Funari M.F., Garcia-Troncoso N. (2024). Environmental impact evaluation of low-carbon concrete incorporating fly ash and limestone. Clean. Mater..

[36] Ferreira R.M., Jalali S., Fernandes A. (2016). Effect of metakaolin on the chloride ingress properties of concrete. KSCE J. Civ. Eng..

[37] Dahanni H., Ventura A., Le Guen L., Dauvergne M., Orcesi A., Cremona C. (2024). Life cycle assessment of cement: Are existing data and models relevant to assess the cement industry’s climate change mitigation strategies? A literature review. Constr. Build. Mater..

[38] Almonayea N., Garcia-Troncoso N., Xu B., Bompa D.V. (2025). Probabilistic embodied carbon assessments for alkali-activated concrete materials. Sustainability.

[39] (2011). Cement—Part 1: Composition, Specifications and Conformity Criteria for Common Cements.

[40] White Star Packed Products (2025). High Strength Cement 52.5R Technical Datasheet. Rev. 1.1. https://www.whitestarpackedproducts.co.uk/_files/ugd/db4c2b_5c0ae53a9815464cb2874274c19aad2d.pdf.

[41] Li N., Unluer C. (2023). Development of high-volume steel slag as cementitious material by ethylenediamine tetraacetic acid induced accelerated carbonation. Powder Technol..

[42] Li N., Unluer C. (2025). Evaluating the recyclability and efficacy of seawater during the wet carbonation of recycled concrete aggregates. Constr. Build. Mater..

[43] Wang X., Cao B., Vlachakis C., Al-Tabbaa A., Haigh S.K. (2023). Characterization and piezo-resistivity studies on graphite-enabled self-sensing cementitious composites with high stress and strain sensitivity. Cem. Concr. Compos..

[44] Baldermann C., Baldermann A., Furat O., Krüger M., Nachtnebel M., Schroettner H., Juhart J., Schmidt V., Tritthart J. (2019). Mineralogical and microstructural response of hydrated cement blends to leaching. Constr. Build. Mater..

[45] Knop Y., Peled A. (2016). Setting behavior of blended cement with limestone: Influence of particle size and content. Mater. Struct..

[46] Abdelmonim A., Bompa D.V. (2021). Mechanical and fresh properties of multi-binder geopolymer mortars incorporating recycled rubber particles. Infrastructures.

[47] AGS Minéraux (2008). ARGICAL-M 1000: Product Specification. Revision 4. https://irp.cdn-website.com/57f31be1/files/uploaded/ARGICAL%20M1000%20-%20Product%20Data.pdf.

[48] (2014). Aggregates for Concrete.

[49] (2012). Tests for Geometrical Properties of Aggregates—Part 1: Determination of Particle Size Distribution—Sieving Method.

[50] (2002). Mixing Water for Concrete—Specification for Sampling, Testing and Assessing the Suitability of Water, Including Water Recovered from Processes in the Concrete Industry, as Mixing Water for Concrete.

[51] Fibre Technologies International Ltd. (2025). Flowaid SCC Technical Datasheet.

[52] (2001). Admixtures for Concrete, Mortar and Grout—Part 2: Concrete Admixtures—Definitions, Requirements, Conformity, Marking and Labelling.

[53] Okamura H., Ouchi M. (2003). Self-compacting concrete. J. Adv. Concr. Technol..

[54] EFCA (2005). The European Guidelines for Self-Compacting Concrete: Specification, Production and Use.

[55] Dhandapani Y., Joseph S., Bishnoi S., Kunther W., Kanavaris F., Kim T., Irassar E., Castel A., Zunino F., Machner A. (2022). Durability performance of calcined clay blended cements—A RILEM TC 282-CCL review. Mater. Struct..

[56] (1999). Methods of Test for Mortar for Masonry—Part 3: Determination of Consistence of Fresh Mortar (by Flow Table).

[57] (2019). Methods of Test for Mortar for Masonry—Determination of Flexural and Compressive Strength of Hardened Mortar.

[58] (2002). Products and Systems for the Protection and Repair of Concrete Structures. Test Methods. Determination of resistance of capillary absorption.

[59] Castro J., Bentz D., Weiss J. (2011). Effect of sample conditioning on the water absorption of concrete. Cem. Concr. Compos..

[60] Hall C. (1989). Water sorptivity of mortars and concretes: A review. Mag. Concr. Res..

[61] Bompa D.V., Xu B., Corbu O. (2022). Evaluation of one-part slag-fly-ash alkali-activated mortars incorporating waste glass powder. J. Mater. Civ. Eng..

[62] Rietveld H.M. (1969). A profile refinement method for nuclear and magnetic structures. J. Appl. Crystallogr..

[63] Pane I., Hansen W. (2005). Investigation of blended cement hydration by isothermal calorimetry and thermal analysis. Cem. Concr. Res..

[64] Stutzman P.E., Feng P., Bullard J.W. (2016). Phase analysis of Portland cement by combined quantitative X-ray powder diffraction and scanning electron microscopy. J. Res. Natl. Inst. Stand. Technol..

[65] Snellings R., Chwast J., Cizer Ö., De Belie N., Dhandapani Y., Durdzinski P., Elsen J., Haufe J., Hooton D., Patapy C. (2018). Report of TC 238-SCM: Hydration stoppage methods for phase assemblage studies of blended cements—Results of a round robin test. Mater. Struct..

[66] Taylor H.F.W. (1997). Cement Chemistry.

[67] (2022). Standard Guide for Examination of Hardened Concrete Using Scanning Electron Microscopy.

[68] Sabău M., Bompa D.V., Silva L.F. (2021). Comparative carbon emission assessments of recycled and natural aggregate concrete: Environmental influence of cement content. Geosci. Front..

[69] Sfikas I.P., Badogiannis E.G., Trezos K.G. (2014). Rheology and mechanical characteristics of self-compacting concrete mixtures containing metakaolin. Constr. Build. Mater..

[70] Lagier F., Kurtis K.E. (2007). Influence of Portland cement composition on early age reactions with metakaolin. Cem. Concr. Res..

[71] Courard L., Darimont A., Schouterden M., Ferauche F., Willem X., Degeimbre R. (2003). Durability of mortars modified with metakaolin. Cem. Concr. Res..

[72] Sowoidnich T., Lothenbach B., Kulik D.A., Scrivener K.L. (2023). The impact of metakaolin on the hydration of tricalcium silicate: Effect of C-A-S-H precipitation. Front. Mater..

[73] Barkat A., Kenai S., Menadi B., Kadri E., Soualhi H. (2019). Effects of local metakaolin addition on rheological and mechanical performance of self-compacting limestone cement concrete. J. Adhes. Sci. Technol..

[74] Jiang G., Rong Z., Sun W. (2015). Effects of metakaolin on mechanical properties, pore structure and hydration heat of mortars at 0.17 w/b ratio. Constr. Build. Mater..

[75] Wild S., Khatib J.M. (1997). Portlandite consumption in metakaolin cement pastes and mortars. Cem. Concr. Res..

[76] Almeida M., Iqbal I., Kasim T., Bin Inqiad W., Besklubova S., Sadrolodabaee P., Nowakowski D.J., Rahman M. (2025). Effect of metakaolin and biochar addition on the performance of 3D concrete printing: A meta-analysis approach. Sustainability.

[77] da Cruz T.V.M., Brandão P.R.G., Henriques A.B. (2022). Characterization and mechanical properties of one-part geopolymer based on a pure metakaolin. REM—Int. Eng. J..

[78] Homayoonmehr R., Ramezanianpour A.A., Mirdarsoltany M. (2021). Influence of metakaolin on fresh properties, mechanical properties and corrosion resistance of concrete and its sustainability issues: A review. J. Build. Eng..

[79] Huang Q., Wang Q., Zhang Z., Shen C., Zhou W., Xu X., Zhu X. (2025). Effect of metakaolin fineness on performance and microstructure of cement-based materials. J. Build. Eng..

[80] Bergold S.T., Goetz-Neunhoeffer F., Neubauer J. (2013). Quantitative analysis of C-S-H in hydrating alite pastes by in-situ XRD. Cem. Concr. Res..

[81] Scrivener K., Snellings R., Lothenbach B. (2015). A Practical Guide to Microstructural Analysis of Cementitious Materials.

[82] Damineli B.L., John V.M. (2012). Developing low CO_2_ concretes: Is clinker replacement sufficient? The need of cement use efficiency improvement. Key Eng. Mater..

